# Relationship between Plasma P-Tau217 and Amyloid PET in Racial and Ethnic Underrepresented Groups (RE-URG) Compared with Non RE-URG in LEARN and A4

**DOI:** 10.14283/jpad.2024.124

**Published:** 2024-07-24

**Authors:** Doris Molina-Henry, O. Langford, M.C. Donohue, R. Raman, P. Aisen, K.A. Johnson, R.A. Rissman, R. Sperling, A4 & LEARN Study Teams

**Affiliations:** 1 Alzheimer's Therapeutic Research Institute, Keck School of Medicine, University of Southern California, San Diego, USA; 2 Center for Alzheimer Research and Treatment, Department of Neurology, Brigham and Women's Hospital, Massachusetts General Hospital, Harvard Medical School, Boston, MA, USA

**Keywords:** PET, P-tau, race, ethnicity, Alzheimer's disease

## Abstract

**Background:**

Individuals from diverse racial and ethnic groups are severely underrepresented in Alzheimer's disease trials in part due to disproportionate biomarker ineligibility. Evidence from recent studies support plasma phosphorylated tau 217 (P-tau217) as an early marker for brain Aβ pathology and a reliable marker in predicting elevated brain amyloid PET in cognitively unimpaired adults.

**Objectives:**

To examine whether the relationship between P-tau217 and 18-F florbetapir PET standard uptake value ratios (SUVR) is influenced by race and ethnicity in the Anti-Amyloid treatment in Asymptomatic Alzheimer's disease (A4) preclinical AD studies.

**Design:**

We conducted a retrospective analysis of A4 clinical trial and the LEARN natural history companion study data to evaluate the relationship between baseline P-tau217 and PET SUVR concentration levels by race and ethnicity.

**Setting:**

The analysis was conducted on samples from participants enrolled across 65 study sites in the United States and Canada.

**Participants:**

Cognitively unimpaired adults aged 65–85 enrolled at North American sites in the A4 preclinical AD trial, pre-dose, (N=1018), and the LEARN (N=480) study. Participants were grouped into 2 categories, racial and ethnic underrepresented group (RE-URG) and non-RE-URG (nRE-URG) based on self-identification.

**Measurements:**

A mixed-effects regression model was fit to determine differences in the relationship between P-tau217 and PET SUVR by race and ethnicity, adjusting for age, and APOE ε4 carrier status.

**Results:**

Results from the linear mixed-effects model support that there was no statistically significant effect of race and ethnicity on the relationship between P-tau217 and PET SUVR.

**Conclusion:**

These findings suggest that the relationship between plasma P-tau217 and PET SUVR is the same across race and ethnicity. Future analyses should corroborate these findings in a larger sample and examine whether plasma P-tau217 reflects the differential amyloid prevalence previously reported for other biomarkers of amyloid.

## Introduction

Alzheimer's disease (AD) trials are challenged by a severe underrepresentation of individuals from certain racial and ethnic groups (1, 2, 3). Some of these groups, including Black or African Americans and Hispanic or Latino are known to be at higher risk of developing all cause dementia (4, 5). Despite this higher risk, individuals from racial and ethnic minoritized groups are severely underrepresented in AD clinical trials. This underrepresentation can threaten the external validity of study findings and jeopardize confidence in the ability of future treatments to have equal benefit and risk profiles across populations. Reports from amyloid lowering trials in adults with “preclinical AD”, individuals who exhibit biomarker evidence of brain amyloid pathology but have not yet experienced clinical impairment, suggest disproportionate ineligibility rates of individuals from racial or ethnic underrepresented groups (RE-URG). This disproportionate ineligibility is often based on cognitive exclusion criteria, or not meeting study biomarker cutpoints for amyloid biomarkers including PET, and more recently plasma (6, 7, 8, 9).

Recently we reported (9) differential plasma Aβ42/40 eligibility rates among AHEAD 3–45 Study participants identifying as Hispanic Black, Hispanic White, Non-Hispanic Asian, Non-Hispanic Black, compared to Non-Hispanic White. All RE-URGs had a higher ineligibility rate on plasma compared to Non-Hispanic White. Subsequent evaluation of PET eligibility indicated that among those plasma eligible there was an equal 50% PET eligibility rate across all groups. These results suggest that plasma screening appropriately excludes participants from amyloid lowering treatment trials, as evidenced by consistent performance in predicting likelihood of PET eligibility. Lower amyloid prevalence in RE-URGs consistent with previous findings (6, 7, 10) may explain differential plasma eligibility rates. Importantly, larger scale pre-screening with robust, highly discriminative plasma biomarkers will be essential to ensure timely and inclusive enrollment of RE-URGs.

There are many advantages of plasma biomarker screening for Alzheimer's disease clinical trials including significant time and cost-savings (11), they are minimally invasive and thus more acceptable than cerebrospinal fluid. Their use is a promising approach to engage populations that are historically underrepresented in AD trials, and to conduct large scale screenings through registries (12) or at community settings. Further, plasma biomarkers may in the future eliminate additional logistical and cost barriers by limiting or eliminating the need for PET. Among currently used biomarkers are the plasma Aβ42/40 ratio, which adjusted across age and apolipoprotein E (APOE) status can reliably predict likelihood of PET eligibility (9). Adjusted Aβ42/40 ratio has been recently superseded by high performing P-tau plasma tests which accurately reflect Aβ plaque pathology even in pre-symptomatic stages in sporadic and familial AD (13). Phosphorylated tau at Threonine 217 (pTau217) has demonstrated superior performance to other pTau biomarkers including pTau181 and pTau231 with areas under the curve (AUC) exceeding 90% in discriminating individuals with evidence of amyloid pathology as measured by PET (14, 15).

While the association between plasma tau species with amyloid PET has been described (16, 17) the impact of race and ethnicity on the association between P-tau217 and amyloid PET on race and ethnicity remains understudied (18, 19). Considering the reported differential prevalence of amyloid in plasma across RE-URGs (9, 20) and the proposed use of blood-based biomarkers in lieu of PET, evaluating whether the relationship between P-tau217 and PET is consistent across all racial and ethnic groups has important implications for differential trial eligibility. Furthermore, comparisons of plasma markers with a reference standard may be a more beneficial approach in establishing consistent performance across RE-URGs than comparison of absolute values (19). We examined the relationship between P-tau217 and 18-F florbetapir amyloid PET standard uptake value ratios (SUVr) by self-reported racial and ethnic groups in the Anti-Amyloid treatment in Asymptomatic in Alzheimer's Disease (A4) Study and the natural history companion study Longitudinal Evaluation for Amyloid Risk and Neurodegeneration (LEARN).

## Methods

### The A4 Study and LEARN

The A4 Study (NCT02008357) is a 240-week, Phase 3 double-blind, placebo-controlled randomized clinical trial. The study enrolled asymptomatic adults ages 65–85 with biomarker evidence of brain amyloid accumulation as measured by amyloid 18-F florbetapir PET. The study aimed to evaluated whether solanezumab, an anti-amyloid antibody treatment could slow cognitive decline (21, 22). Interested, recruited individuals 65–85 years of age, with a study partner, living independently without dementia or mild cognitive impairment were consented and invited for in-clinic screening visits to establish eligibility. The screening process and eligibility criteria have been published in detail elsewhere (22, 23). Briefly, individuals with Clinical Dementia Rating (CDR) global score of 0, Mini-Mental State Examination (MMSE) of 25–30, and Logical Memory delayed Recall (LMDR IIA) score of 6–18 were eligible to proceed to 18-F florbetapir PET.

The LEARN Study, a natural history observational arm of the A4 Study was comprised of individuals who matched A4 participants on all screening criteria, but screen failed due to subthreshold levels of amyloid accumulation on PET imaging. The LEARN cohort was run in parallel to the treatment arm and underwent identical cognitive assessments every six months, with imaging and biomarker outcomes collected at the end of the study.

### Biomarkers

#### Amyloid PET imaging

Participants eligible for PET imaging underwent 18-F florbetapir scans. Participants were randomized to A4 if determined to have evidence of elevated amyloid accumulation, as determined by an algorithm that combined visual read and mean cortical standardized uptake value ratio (SUVr). Elevated amyloid was primarily defined by a quantitative SUVr of 1.15 (24), a SUVr of 1.10 to >1.15 was considered elevated only when a visual read was deemed positive by a two-reader consensus determination. Additional details of the screening, randomization algorithm and trial results are reported elsewhere (21, 23).

### Plasma Biomarker assay

P-tau217 was quantified on analytically validated ECL immunoassay using a MesoScale Sector S Imager 600 MM at CAP-accredited, CLIA-certified (Lilly Clinical Diagnostics Laboratory). Samples included in this analysis were collected within +/−215 days of PET imaging. Samples for A4 were collected on average 60 days following PET imaging but prior to dosing. LEARN samples were collected on average 30 days prior to PET imaging (Supplement: Table 1).

### Sample and Statistical Analyses

Self-reported race and ethnicity was collected for randomized A4 and LEARN. Race was collected as “American Indian or Alaskan Native,” “Asian,” “Black or African American,” “More than one race” “Unknown or not reported” and “White”. Ethnicity was captured as “Hispanic or Latino,” “Not Hispanic or Latino,” “Unknown or Not Reported”. Participants historically underrepresented in AD trials in North America were grouped under one single group and classified as RE-URG, given the smaller number of those subpopulations. Self-identified “Non-Hispanic” and “White” participants were classified as non-RE-URG (nRE-URG).

We summarized participant characteristics using means and standard deviations for continuous variables, with categorical variables summarized using counts and percentages. To evaluate whether there was an influence of race and ethnicity in the relationship between P-tau217 and PET SUVr we fit a linear mixed-effects regression model to amyloid PET SUVr, adjusting for fixed-effects of age, APOE ε4 carrier status, P-Tau217, race and ethnicity, and interaction of P-tau217 and race and ethnicity. A random-intercept model was chosen to account for the between-site variability observed. The analysis was restricted to a subset of participants enrolled in North America, including the 60 sites in the United States and 4 sites in Canada. Model diagnostics suggested the violation of model assumptions (Supplement Figure S1). To have valid model assumptions including normality of residuals, homoscedasticity of residuals and independently, identically distributed errors, we performed Boxcox transformations of amyloid PET SUVr and plasma P-tau217 (1/√SUVR 1/√pTau217). Transformation details for SUVr and P-tau217 included in Supplement Figure S2A, B. An exploratory analysis was performed to assess whether the Non-Hispanic Black (NHB) P-tau217/PET SUVr relationship differed from nRE-URG. As there are many more nRE-URG individuals compared to NHB participants in our sample, with differing amyloid prevalence levels, we matched pairs on age, APOE ε4 carrier status and P-tau217 using an algorithm developed by Ho et al., 2007 (25), to reduce bias in our assessment. For this analysis we kept the same fixed-effects as mentioned above, however with a much smaller sample size, the random-intercept was reduced to a fixed-effect. For visualization purposes data are shown in their original scales and all analyses were conducted using R version 4.4.0.

## Results

1498 A4 and LEARN participants (A4 N=1018 and LEARN N=480) were enrolled at North American sites. Demographic characteristics by study arm are represented in the Supplement Table S1). Demographic characteristics for RE-URG and nRE-URG across all arms are represented in Table 1. Out of the 1498 in the sample, 134 participants were classified as RE-URG and 1364 were nRE-URG. The RE-URG was comprised of 7 (5.2%) American Indian or Alaskan Native, 17 (1.1%) Asian, 36 (26.9%) Black or African American, 13 (9.7%) More than one race, 8 (6%) Unknown or not reported and 54 (39.6%) White race, 47 (35.1%) Hispanic or Latino, 73 (54.5%) Not Hispanic or Latino, 14 (10.4%) were Unknown or Not Reported. Both groups were comparable in age, overall mean 71.48 (4.73), and mean years of education 16.68 (2.68). Both groups had a higher proportion of females (overall 60.8%) compared to males (overall 39.2%). The nRE-URG had a greater proportion of APOE ε4 carriers, n=659 (48%), compared to the RE-URG, n=47 (35%; Table 1) driven by a greater proportion of nRE-URG eligible for the A4 Study.

**Table 1:** Demographic characteristics of participants enrolled in North America by group

Characteristic	nRE-URG (N=1364)	RE-URG (N=134)	Total (N=1498)
Age			
Mean (SD)	71.42 (4.72)	72.11 (4.83)	71.48 (4.73)
Range	65.00–85.74	65.04–85.60	65.00–85.74
Sex			
Male (%)	541 (39.7)	46 (34.3)	587 (39.2)
Female (%)	823 (60.3)	88 (65.7)	911 (60.8)
Education			
Mean (SD)	16.70 (2.63)	16.49 (3.14)	16.68 (2.68)
Range	7.00–30.00	10.00–30.00	7.00–30.00
Hispanic, Latino/a, or Spanish origin?			
Hispanic or Latino (%)	0 (0.0)	47 (35.1)	47 (3.1)
Not Hispanic or Latino (%)	1364 (100.0)	73 (54.5)	1437 (95.9)
Unknown or not reported (%)	0 (0.0)	14 (10.4)	14 (0.9)
Race			
American Indian or Alaskan Native (%)	0 (0.0)	7 (5.2)	7 (0.5)
Asian (%)	0 (0.0)	17 (12.7)	17 (1.1)
Black or African American (%)	0 (0.0)	36 (26.9)	36 (2.4)
More than one race (%)	0 (0.0)	13 (9.7)	13 (0.9)
Unknown or not Reported (%)	0 (0.0)	8 (6.0)	8 (0.5)
White (%)	1364 (100.0)	53 (39.6)	1417 (94.6)
APOE			
Carrier (%)	659 (48)	47 (35)	706 (47)
Non-carrier (%)	703 (52)	87 (65)	790 (55)
Unknown	0		
SUVr			
Mean (SD)	1.23 (0.22)	1.18 (0.21)	1.22 (0.22)
Range	0.79–2.09	0.82–1.86	0.79–2.09
Centiloid			
Mean (SD)	47.45 (40.57)	38.00 (37.64)	46.60 (40.40)
Range	−32.63–205.36	−27.14–163.25	−32.63–205.36
P-tau217 (U/mL)			
Mean (SD)	0.24 (0.15)	0.23 (0.17)	0.24 (0.15)
Range	0.08–1.50	0.08–1.26	0.08–1.50
Treatment arm			
LEARN (%)	431 (31.6)	49 (36.6)	480 (32.0)
Placebo (%)	472 (34.6)	44 (32.8)	516 (34.4)
Solanezumab (%)	461 (33.8)	41 (30.6)	502 (33.5)
Days between Ptau217-PET			
Mean (SD)	33.89 (49.26)	28.93 (50.17)	33.45 (49.34)
Range	−103.00–215.00	−84.00–181.00	−103.00–215.00
Country			
USA (%)	1321 (96.8)	129 (96.3)	1450 (96.8)
Canada (%)	43 (3.2)	5 (3.7)	48 (3.2)

**Figure 1:**
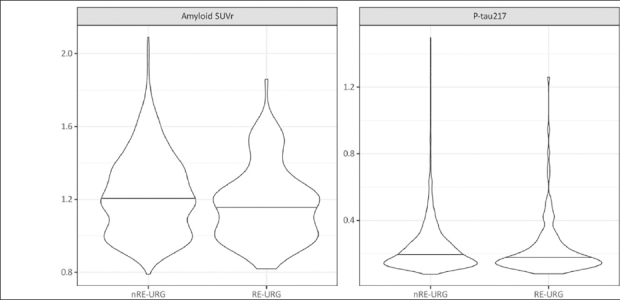
Baseline amyloid PET SUVR and P-tau217 distributions by race and ethnic group Racial and ethnic underrepresented group (RE-URG) and non-RE-URG (nRE-URG).

**Figure 2:**
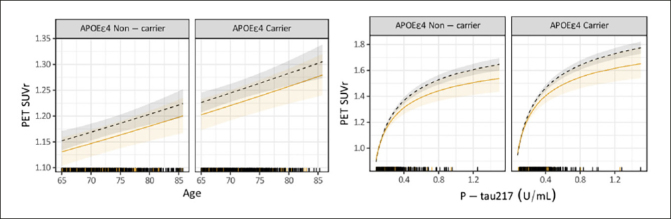
Relationship of continuous variables, age and P-tau217, to PET SUVr by race and ethnic underrepresented group (RE-URG) and non-RE URG (nRE-URG) Shading reflects 95% confidence interval for each curve

P-tau217 levels were the similar in both groups, RE-URG mean=0.23 (0.17) U/mL vs nRE-URG mean=0.24 (0.15) U/mL, mean amyloid PET was lower in RE-URG (38 (37.64) centiloid (CL)); SUVr of 1.18 (0.21)), than in nRE-URG (47.45 (40.57) CL; SUVr of 1.23 (0.22). Distribution of P-tau217 and PET SUVr across RE-URG and nRE-URG can be observed in Figure 1. Notably, while the distributions are similar, PET SUVR median is slightly lower in RE-URG than in nRE-URG. Further, we examined the relationship between P-tau217 and PET SUVr by race and ethnicity using a linear mixed-effects regression model described above. Results from the model suggest that while there is a trend toward statistical significance by race and ethnicity on PET SUVr (p=0.084), there is no statistically significant influence of race and ethnicity on the relationship P-tau217 and PET SUVr (p=0.160, Table 2). Figure 2 depicts a potential similar relationship between PET SUVr and age, and between PET SUVr and P-tau217, treating age and P-tau217 as continuous variables, in APOE ε4 carriers vs non-carriers.

**Table 2:** Covariate estimates from the linear mixed-effects model predicting amyloid PET SUVr adjusting for racial and ethnic underrepresented group (RE-URG) age, APOE, and P-tau217 and race and ethnicity

Covariate	Estimate	95% CI	p-value
(Intercept)	0.79	(0.75, 0.84)	<0.001
Age	−1.34e-03	(−1.93e-03, −7.49e-04)	<0.001
1/√(P-tau217)	0.10	(0.09, 0.11)	<0.001
APOEε4 Carrier	−0.03	(−0.03, −0.02)	<0.001
RE-URG	0.04	(−4.98e-03, 0.08)	0.084
1/√(P-tau217):RE-URG	−0.01	(−0.03, 5.01e-03)	0.160


Note: a. Number of observations in the model: 1496

**Figure 3:**
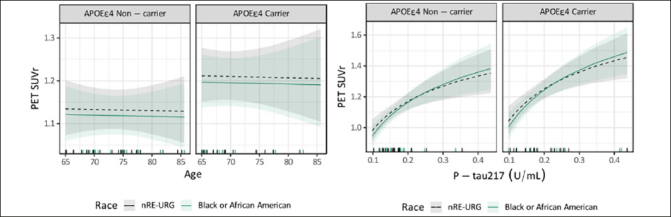
Relationship between PET SUVR and continuous variables age and P-Tau217 in Non-Hispanic Black (NHB) and non-racial and ethnic underrepresented group (nRE-URG)

Among NHB adults (n=36) and nRE-URG adults (n=36) included in the exploratory subset analysis, age, APOE ε4 carrier status and P-tau217 were similar across groups as these were used in the matching pairs algorithm. This resulted in average age of 72, 42% APOE ε4 carriers and average P-tau217 of 0.19 U/mL in both groups. Amyloid PET was 36.07 (29.89) CL for NHB and 39.17 (32.64) CL for nRE-URG, which corresponded to 1.17 (0.16) and 1.18 (0.19) SUVr. The relationship between P-tau217 and PET SUVr was the same for both NHB and nRE-URG, see Figure 3. In this subset, results from the linear mixed effects model on PET SUVr indicate that there was no statistically significant difference between NHB and nRE-URG on PET SUVr (p=0.638), nor was there a statistical significance on the relationship between P-tau217 and PET SUVr by category (p=0.579).

## Discussion

We examined differences in the relationship between P-tau217 and PET SUVr levels across individuals who identified as belonging to RE-URGs and nRE-URGs in the preclinical AD population enrolled in the A4 Study and the observational LEARN cohort. In our analysis, race and ethnicity did not influence the relationship between P-tau217 and PET SUVr. Our results suggest that when compared to PET amyloid, as a reference standard, P-tau217's predictive ability is consistent across racial and ethnic groups. Additionally, these findings were consistent in an exploration of a small subgroup of participants who identified as Non-Hispanic Black compared to nRE-URG, suggesting equal performance of P-tau217 in Non-Hispanic Black adults compared to RE-URG.

Previously, we evaluated rates of PET eligibility of individuals who met the plasma cutpoint in another AD preclinical AD trial (AHEAD 3–45 Study), using a different plasma assay (C2N mass spec Aβ42/40) and a different amyloid PET tracer (18-F-NAV 6240). Similar to the current results, we found that rates of eligibility by PET as predicted by plasma were equal across racial and ethnic groups. Our finding supports the consistent performance of plasma markers across race and ethnicity in establishing likelihood of amyloid PET positivity. The current analysis replicates these findings by evaluating the differences in relationship between P-tau217 and amyloid PET in individuals who identified as belonging to one of several racial or ethnic underrepresented groups in the A4 Study. Although preliminary, given the smaller size of the RE-URG population, these results have important implications for the use of P-tau217, as a screening blood-based biomarker, for more inclusive trial enrollment. This finding is encouraging in light of the use of P-tau217 as a screening biomarker at the earliest stages of screening in the AHEAD 3–45 clinical trials program. Furthermore P-tau217 may be a promising biomarker for a more inclusive approach to screening in future preclinical AD trials.

Race and ethnicity are social constructs with no biological basis. Biological differences often attributed to these constructs are likely due to socioeconomic disadvantage disproportionately experienced by some racial and ethnic groups. NH Black adults are at twice the risk of developing dementia and are less likely to be eligible for AD clinical trials. Given these paradoxical findings, we conducted an exploratory analysis with a subset of NHB adults and nRE-URG matched on age, APOE, P-tau217, with equal years of education. Results suggest that relationship between P-tau217 and PET SUVr in both groups was the same. While this is a small sample, this result is consistent with previous results in NHB adults screened in the AHEAD 3–45 Study, based on Aβ42/40 plasma amyloid levels. Further examination in a larger sample with greater representation of NHB adults will be critical to bolster these findings and corroborate whether racial differences in amyloid prevalence underlie underrepresentation of this and other racial groups in AD clinical trials.

Plasma based biomarkers have gained much attention as a potential diagnostic tool in clinical settings and a powerful screening tool in preclinical AD studies (26). Their lesser invasiveness and lower cost compared to PET increases their potential for facilitating large-scale screening inclusive of populations traditionally underrepresented in AD trials. However, disproportionate exclusion of individuals from underrepresented racial and ethnic backgrounds have prompted questions and concerns around differential performance across groups and the potential need for different cutpoints. Previously, we reported on consistency in performance of plasma Aβ42/40 as it relates to amyloid PET in appropriately excluding individuals who did not meet the amyloid threshold for randomization in the AHEAD 3–45 Study (9). Here we report consistency in findings with P-tau217, a superior marker of plasma amyloid due to its strong association with amyloid burden by PET in early accumulating brain regions and its association with longitudinal increase (14, 27, 28). These findings further support the use of standard plasma cutpoints for the determination of amyloid eligibility in preclinical AD studies across race and ethnicity, but highlight the need for further research to elucidate the underpinnings of potential differences in amyloid prevalence.

### Limitations

The analyses presented in this article are not without limitations. The numbers of individuals across RE-URG populations in A4 and LEARN were small, and as such subpopulation analyses are prohibitive, and prompting the grouping under a single RE-URG category. Of note, while we explored in a subgroup of NH Black participants matched on age, APOE and P-tau217 and who had average equal number of years of education the sample of NH Black in the study was relatively small and limits the conclusions that can be drawn related to this specific group. Future analyses in cohorts with larger, more inclusive samples, and biomarker data, such as the AHEAD 3–45 and APEX Study cohorts, will allow evaluation of this relationship across subpopulations.

*Funding:* A4 and LEARN were supported by a public-private-philanthropic partnership which included funding from the National Institute of Aging of the National Institutes of Health (R01 AG063689, U19AG010483 and U24AG057437), NIA ACTC U24 AG057437 to PSA and RAS]. Additionally, the studies were supported by Eli Lilly and the Alzheimer's Association. Open access funding provided by SCELC, Statewide California Electronic Library Consortium.

*Ethical Considerations:* Approval from an institutional review board or ethics committee was obtained at each of the sites. All participants and their study partners provided written informed consent prior to data collection, which included consent for data sharing.

*Conflicts of interest:* DMH: has received research support from the American Heart Association and the Alzheimer's Association, American Heart Association and GHR Foundation. OL: Nothing to report. MCD: has received research funding from the National Institutes of Health, Janssen, Eli Lilly, and Eisai, reports consulting fees from Roche and his spouse is a full-time employee of Janssen. RR: has received research support from the National Institutes of Health (NIH), the Alzheimer's Association, American Heart Association, Eli Lilly and Eisai. PSA: has received grants or contracts from the National Institutes of Health (NIH), Alzheimer's Association, Foundation for NIH (FNIH), Lilly, Janssen and Eisai and consulting fees from Merck, Biogen, AbbVie, Roche, and Immunobrain Checkpoint. KAJ: has received research funding from National Institutes on Aging, National Institutes of Health, Alzheimer's Association, and the GHR Foundation. He has served as a consultant for Merck, Novartis, Janssen, and Prothena. RAR: has research support from the National Institute on Aging, the Alzheimer's Association and is a consultant for Amydis Inc, Bioivt, Lexeo, Keystone Bio, Allyx, DiamiR, Ionis and PrecisionMed. RAS: reports grant support from the National Institutes on Aging, National Institutes of Health, Alzheimer's Association, GHR Foundation, and Gates Ventures. She has received trial research funding from Eisai and Eli Lilly for public-private partnership trials. She reported serving as a consultant for AbbVie, AC Immune, Alector, Biohaven, Bristol-Myers-Squibb, Ionis, Janssen, Genentech, Merck, Prothena, Roche, and Vaxxinity.
